# Prediction of pandemic risk for animal-origin coronavirus using a deep learning method

**DOI:** 10.1186/s40249-021-00912-6

**Published:** 2021-10-24

**Authors:** Zheng Kou, Yi-Fan Huang, Ao Shen, Saeed Kosari, Xiang-Rong Liu, Xiao-Li Qiang

**Affiliations:** 1 Institute of Computing Science and Technology, Guangzhou University, Guangzhou, 510006 China; 2 Department of Computer Science, Xiamen University, Xiamen, 361005 China

**Keywords:** Coronavirus, Pandemic risk, Viral genome, Deep learning

## Abstract

**Background:**

Coronaviruses can be isolated from bats, civets, pangolins, birds and other wild animals. As an animal-origin pathogen, coronavirus can cross species barrier and cause pandemic in humans. In this study, a deep learning model for early prediction of pandemic risk was proposed based on the sequences of viral genomes.

**Methods:**

A total of 3257 genomes were downloaded from the Coronavirus Genome Resource Library. We present a deep learning model of cross-species coronavirus infection that combines a bidirectional gated recurrent unit network with a one-dimensional convolution. The genome sequence of animal-origin coronavirus was directly input to extract features and predict pandemic risk. The best performances were explored with the use of pre-trained DNA vector and attention mechanism. The area under the receiver operating characteristic curve (AUROC) and the area under precision-recall curve (AUPR) were used to evaluate the predictive models.

**Results:**

The six specific models achieved good performances for the corresponding virus groups (1 for AUROC and 1 for AUPR). The general model with pre-training vector and attention mechanism provided excellent predictions for all virus groups (1 for AUROC and 1 for AUPR) while those without pre-training vector or attention mechanism had obviously reduction of performance (about 5–25%). Re-training experiments showed that the general model has good capabilities of transfer learning (average for six groups: 0.968 for AUROC and 0.942 for AUPR) and should give reasonable prediction for potential pathogen of next pandemic. The artificial negative data with the replacement of the coding region of the spike protein were also predicted correctly (100% accuracy). With the application of the Python programming language, an easy-to-use tool was created to implements our predictor.

**Conclusions:**

Robust deep learning model with pre-training vector and attention mechanism mastered the features from the whole genomes of animal-origin coronaviruses and could predict the risk of cross-species infection for early warning of next pandemic.

**Graphical Abstract:**

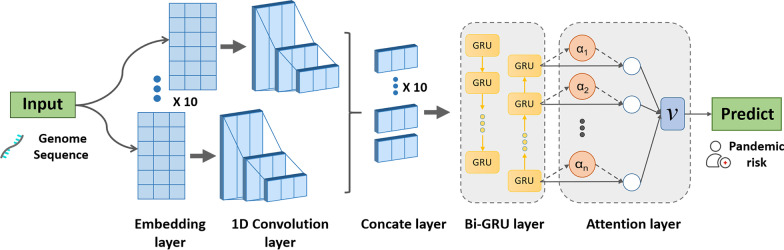

**Supplementary Information:**

The online version contains supplementary material available at 10.1186/s40249-021-00912-6.

## Background

Coronaviruses (CoV) are a group of RNA viruses whose linear, positive-sense, single-stranded RNA genomes are the longest among the known RNA viruses. The International Committee on Taxonomy of Viruses (ICTV) classifies coronaviruses into α, β, γ, and δ genera [1]. Seven types of coronaviruses that infect humans have been identified: human coronavirus (HCoV) 229E, OC43, NL63, and HKU1; severe acute respiratory syndrome coronavirus (SARS-CoV and SARS-CoV-2); and Middle East respiratory syndrome coronavirus (MERS-CoV). SARS-CoV, MERS-CoV, and SARS-CoV-2 are highly contagious and have caused three epidemics or pandemics this century [2, 3].

The coronaviruses responsible for pandemics are animal-origin pathogens transmitted to humans through the intermediate host [4–7]. The intermediate hosts of SARS-CoV and MERS-CoV were civets and dromedaries, respectively [8, 9]. The direct host of SARS-Cov-2 is not clear but is closely related to bats and pangolins [10, 11]. Coronaviruses are capable of cross-species infection through accumulation of point mutation and recombination of their RNA genome [2]. All coronaviruses responsible for epidemics or pandemics come from wild animals, are spread through respiratory droplets and close contact, and can cause severe pneumonia. Every outbreak of the coronavirus with the novelty of viral antigens has caused severe economic and societal damage. Consequently, we urgently need to develop a prediction model of the pandemic risk for human coronavirus infection and improve the prevention and control of infectious diseases for next pandemics.

The spike protein on the surface of virus particle is the most important surface membrane protein of coronaviruses, being responsible for their binding to the host cell membrane receptor and membrane fusion. It plays a very important role in cross-species infection [12]. The adaptation of other viral proteins to the internal environment of new host also affects viral replication [13]. These facts need to be considered when modeling viral infection, and artificial genome data should be used to increase the weight of the spike protein and build a robust model.

Deep learning developed rapidly in recent years, which has triggered changes in application fields such as speech recognition, image understanding, natural language processing (NLP). A recurrent neural network (RNN) is a neural network used to process sequence data and has the ability to capture the inherent characteristics of time series [14]. The original RNN model is affected by problems of gradient disappearance or explosion, proposing the long short-term memory (LSTM) network and gated recurrent unit (GRU) network, respectively [15, 16]. At present, RNN and its variants have achieved great success in speech recognition and text translation [17]. Because genomes are also long chains comprising four alphabet units, RNNs can learn and extract the features of biological sequences. A bidirectional GRU was constructed to predict the binding preference of RNA and protein [18]. An RNN combined with an attention mechanism to predict enhancer-promoter interactions in human genes and achieved good performance [19].

The pandemic risk for animal-origin coronavirus is closely related to variations within the viral genome. We used the natural language model to construct a prediction model based on the phenotype of infection, named Coronavirus Cross-species Infection with Deep Learning (CCSI-DL), which models and analyzes six types of coronaviruses. It uses a one-dimensional (1D) convolution to extract the local features of the sequences, a GRU network to extract the long-term dependence of the sequence in two directions, and an enhanced attention mechanism to capture the weight of key features [19]. The model predicts the cross-species infection risk of animal-origin coronaviruses with well performance and can be used for early warning of pandemic risk.

## Methods

### Initial virus data

Coronavirus sequences were accessed from the Coronavirus Genome Resource Library (https://ngdc.cncb.ac.cn/ncov/) on June 30, 2020, including those of MERS-CoV, HCoV-OC43, HCoV-NL63, HCoV-229E, HCoV-HKU1, SARS-CoV (combined SARS-CoV-1 and SARS-CoV-2) genome sequences and animal-origin coronaviruses [20]. A total of 3257 genomes were downloaded and human- and animal-origin coronaviruses were regarded as positive and negative samples, respectively (see Additional file 1). Using the *K*-nearest neighbors algorithm (*k* = 5), the negative samples (animal-origin coronaviruses) were divided according to the six types of positive samples (Quad-nucleotide frequency as features; Six human groups: MERS-CoV, HCoV-OC43, HCoV-NL63, HCoV-229E, HCoV-HKU1, SARS-CoV), then combined with the corresponding positive samples to form six types of coronavirus data sets (see Additional file 2).

### Artificial negative data

The spike protein is the most important surface membrane protein of coronaviruses and is responsible for their binding to the receptor of host cell, which plays the key role in transmission efficiency and host range [13]. If the spike protein of positive virus is replaced with that of negative virus (see Additional file 3), the positive virus should significantly decrease transmission efficiency and its phenotype label should be changed [13]. According to the strategy of artificial recombination in silicon, the generated sequences based on the replacement of the coding region of the spike protein for the initial positive sample were added to the negative sample data set (see Additional file 4). During training, the weights of the spike protein were further increased in the model, thereby improving the prediction accuracy with robustness. Considering the synergistic effect of other viral proteins on cross-species infection, this approach is consistent with biological studies of host adaption [13]. After the addition of artificial negative data and the balance of sample number (direct duplication), the final dataset of six viruses was shown in Table 1.

**Table 1:** Genomic data for six coronaviruses

	Positive samples, *n*	Negative samples, *n*
MERS-CoV	1044	1138
HCoV-OC43	482	534
HCoV-NL63	240	262
HCoV-229E	240	262
HCoV-HKU1	390	387
SARS-CoV	638	587

### Framework of the CCSI-DL model

**Fig. 1:**
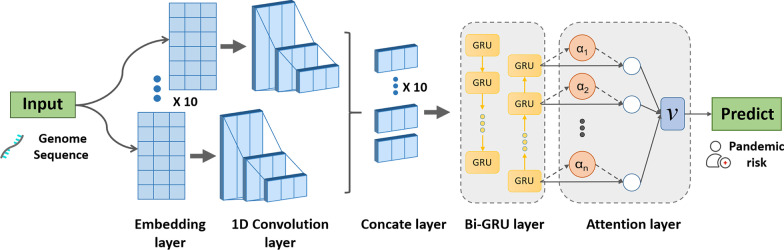
Flowchart of the deep learning method. The Coronavirus Cross-species Infection with Deep Learning (CCSI-DL) model consists of five main steps: genome segmentation, sequence embedding, one-dimensional convolution, the recurrent neural network, and the attention mechanism

The CCSI-DL model consists of five main steps: genome segmentation, sequence embedding, 1D convolution, the RNN, and the attention mechanism. Figure 1 shows the structure of the proposed model in the paper.

### Sequence segmentation and embedding

Genome sequence of coronavirus cannot be directly used for model construction. The method of traditional sequence conversion uses one-hot vectors to encode DNA sequence fragments. Because the information between each vector is independent, the model cannot capture the hidden associated information in the sequence and is therefore unsuitable for deep learning algorithms. To avoid this problem, we used the DNA vector obtained by the dna2vec method [21] as the pre-training vector. The dna2vec method is based on the word2vec word embedding model [22], which is the classical text representation method in the NLP field.

The dna2vec method uses DNA sequence fragments of length *k* (*k*-mers) as words. Its purpose is to calculate the distributed representation of DNA fragments and capture the associated information in the original sequence. Based on the pre-training vector, the model uses fine-tuning strategies in the embedding layer to improve the performance of the model [23]. In the paper, we chose *k* = 2 and an embedding dimension of 8 [19].

The length of the original genome sequence of the coronavirus is about 27–32 kb. We took a two-base RNA fragment as a basic word and preprocessed the original genome sequence to obtain a numerical index sequence. To facilitate model input, the obtained index sequence was rounded to a length of 30 kb and divided into 10 equal segments (3 k for each segment). The embedding layer of the model performed embedding of the input sequence based on the pre-trained vector. To improve the performance of the model, the weight of the embedding layer was set to be trainable so that the DNA vector could be fine-tuned according to the training data of coronavirus genome.

### One-dimensional convolution

The CCSI-DL model combines a 1D convolution with an RNN for feature extraction. A 1D convolution is firstly used to capture the local correlation features in the sequence and then input into the bidirectional GRU network to extract the global correlation features. The 1D convolution slides along the data in one dimension and extracts features from shorter segments in the genome sequence.

For computer vision tasks, using a deeper 2D convolution model can produce a more accurate classification [24], but increasing the network depth does not necessarily improve performance for 1D data [25]. The 1D convolutional layer is used to extract the local features of the sequence after embedding. We set the number of convolution kernels (filters) to 4 and the length of convolution kernels (kernel_size) to 50, and used the rectified linear activation function (ReLU). The ReLU can give the sparsity for the network and the pooling layer can reduce the computational complexity. The maximum pooling layer was connected after the convolutional layer to further reduce the sequence length, increase the calculation speed, and avoid overfitting.

### Recurrent neural network

The recurrent unit used in the RNN was the GRU network, which is improved by the LSTM network and can also solve problems such as long-term memory and gradients in back propagation [26]. The GRU removes the cell state from the LSTM network and retains two gated units (the update gate and the rest gate), which simplifies the LSTM structure and reduces training complexity while achieving a similar experimental effect.

To capture characteristics of biology sequences adequately, a bidirectional GRU architecture was used (two unidirectional GRU layers, one forward and one backward). This architecture connects the forward hidden state and the reverse hidden state, enabling the output result to simultaneously account for the sequence correlation of the forward and backward states. The output of a single GRU layer was set to 50 dimensions, and the two opposing outputs were connected to obtain a total output dimension of 100.

### Attention mechanism

Following the bidirectional GRU layer, the attention layer is used to learn the weights of features. Inspired by human attention, its purpose is to focus attention on a specific part based on a large amount of information. The attention mechanism was first applied in the field of image processing, then introduced into the NLP field for machine translation in 2015 [27] and extended to various other NLP tasks, resulting in many improvements [28]. Assuming that the features in the sequence are not equally important to the prediction, using the attention mechanism can improve the contribution of key features to the prediction. The attention mechanism is described by the following formulae [19]:

$${h}_{i}=\mathrm{tan}h({W}_{\omega }{f}_{i}+{b}_{\omega })$$

$${\alpha }_{i}= \frac{\mathrm{exp}({h}_{i}^{T}{h}_{\omega })}{{\sum }_{i}\mathrm{exp}({h}_{i}^{T}{h}_{\omega })}$$

$$v=\sum_{i}{\alpha }_{i}{f}_{i}$$

For the *i*th feature *f*_*i*_ output by the previous layer, its hidden representation *h*_*i*_ is calculated. The importance of features is measured by the similarity between *h*_*i*_ and the context vector *h*_*w*_. The normalized weight *α*_*i*_ of each feature is obtained by multiplying *h*_*w*_ by *h*_*i*_ and then using the softmax function. The feature vector *f*_*i*_ is multiplied by the corresponding weight *α*_*i*_ and summed to the final output vector *v* used for prediction. *W*_*ω*_, *b*_*ω*_, and *h*_*ω*_ were randomly initialized and learned during model training.

### Model training and evaluation

During training, we used a batch size of 24, the cross-entropy loss function, and the Adam optimization algorithm to update the network weights. To avoid overfitting, a batch normalization layer and a dropout layer (random dropout probability = 0.5) were added after the merge layer. After amplification of positive samples, the six virus data sets were randomly divided into a training set (90%) and a test set (10%). The model was trained using the training set for 15 rounds, then the effect of the model training was evaluated using the test set.

To evaluate the predictive models, we calculated the area under the receiver operating characteristic curve (AUROC) [29] and the area under precision-recall curve (AUPR) [30], both of which are suitable for imbalanced data sets. The receiver operating characteristic (ROC) curve plots the true positive rate as a function of the false positive rate. The closer the AUROC is to 1, the better the performance of the model. The ROC curve is not affected by the distribution of positive and negative samples, so AUROC is suitable as an evaluation index for an unbalanced binary classification model. The precision-recall curve plots the precision as a function of the recall, reflecting the trade-off between the model's accuracy in identifying positive examples. The closer the AUPR value is to 1, the better the performance of the model.

To verify the advantage of pre-trained DNA vector and attention mechanism on the performance of CCSI-DL-specific and CCSI-DL-general, three variant models were proposed: (1) CCSI-DL-nopre, in which the embedding layer does not use pre-training DNA vectors; (2) CCSI-DL-onehot, which does not use an embedding layer and only uses a one-hot-coded sequence as input; (3) CCSI-DL-noatt, which does not use the attention layer. The same training processes were applied to specific and general models: CCSI-DL-spe-nopre, CCSI-DL-spe-onehot, CCSI-DL-spe-noatt, CCSI-DL-gen-nopre, CCSI-DL-gen-onehot, and CCSI-DL-gen-noatt.

### Implementation of the prediction tool

We used the Python 3.7.4, tensorflow 2.1.0 and Keras 2.3.1 to create an easy-to-use tool that implements our predictor, which is freely accessible via https://github.com/kouzheng/CCSI-DL and can run in an end-to-end way and handle massive data. Users need to prepare the query sequences of coronavirus genome in the FASTA format, input the name for query file, and set confidence parameter (from 0.0 to 1.0) before running the tool. Setting a smaller confidence parameter results in more sensitive predictions. A predicted phenotype of pandemic risk is labeled “H”, while the label “N” indicates no transmission.

## Results

### Specific model for each virus group

As there are genetic differences in the sequences of the six virus types, we created separate prediction models using the genome data set for each of the six viruses (called CCSI-DL-specific) and evaluated the generalizability of each specific model to other virus groups. The AUROC and AUPR values obtained after training the six specific models were shown in Table 2. The best results (1 for AUROC and 1 for AUPR) were obtained when the model was trained and tested using the same group of virus data set, while the predictive performances using test data sets from different virus groups was reduced significantly. The MERS-CoV model achieved well performance for the MERS-CoV data (1 for AUROC and 1 for AUPR) and bad prediction for the HCoV-HKU1 data (0.03 for AUROC and 0.333 for AUPR), the HCoV-NL63 data (0.335 for AUROC and 0.375 for AUPR). The SARS-CoV model had excellent performance for the SARS-CoV and MERS-CoV data and low prediction for the HCoV-OC43 data (0.946 for AUROC and 0.887 for AUPR), HCoV-NL63 data (0.96 for AUROC and 0.881 for AUPR) and HCoV-229E data (0.863 for AUROC and 0.734 for AUPR).

**Table 2:** Prediction performance of CCSI-DL-specific model for pandemic risk

Train\Test	MERS-CoV		HCoV-OC43		HCoV-NL63		HCoV-229E		HCoV-HKU1		SARS-CoV	
	AUROC	AUPR	AUROC	AUPR	AUROC	AUPR	AUROC	AUPR	AUROC	AUPR	AUROC	AUPR
MERS-CoV	1	1	0.566	0.474	0.335	0.375	0.503	0.441	0.03	0.333	0.984	0.942
HCoV-OC43	0.066	0.328	1	1	1	1	0.528	0.454	0.943	0.896	0.331	0.396
HCoV-NL63	0.262	0.375	1	1	1	1	0.644	0.523	0.895	0.815	0.549	0.488
HCoV-229E	0.516	0.471	0.917	0.808	0.995	0.995	1	1	0.173	0.366	0.79	0.645
HCoV-HKU1	0.432	0.436	0.914	0.924	1	1	0.66	0.534	1	1	0.621	0.536
SARS-CoV	1	1	0.946	0.887	0.96	0.881	0.863	0.734	1	1	1	1

*AUROC* area under the receiver operating characteristic curve, *AUPR* area under precision–recall curve, *CCSI-DL* coronavirus cross-species infection with deep learning

### General model for all virus groups

Training specific models for six coronavirus data sets is time-consuming and has poor generalizability, hence we need to build a general model for all virus groups. We trained a general model, called CCSI-DL-general, by combining the training data of six virus groups and was then evaluated for each virus group. The model was trained using the mix training data set for 15 rounds, then the performance of the general model was evaluated using the test set. As a result, the CCSI-DL-general model provided very good predictions for all of virus groups with the artificial negative data (1 for AUROC and 1 for AUPR).

### Advantage of pre-training vector and attention mechanism

**Fig. 2:**
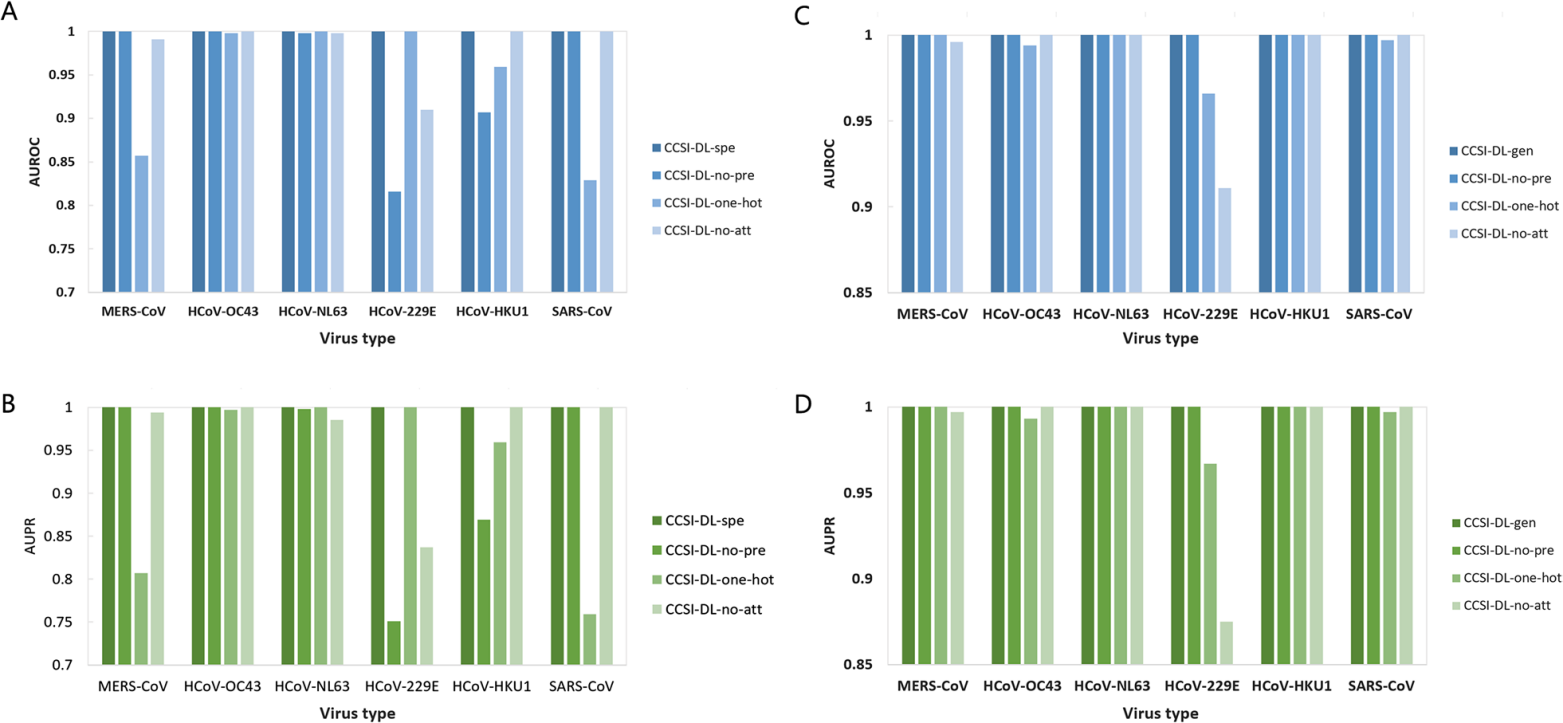
Performance of CCSI-DL-specific and CCSI-DL-general for six virus data sets. **A** Area under the receiver operating characteristic curve (AUROC) and **B** area under the precision-recall curve (AUPR) for CCSI-DL-specific model after removing pre-training vector, using one-hot as input and removing the attention mechanism. **C** AUROC and **D** AUPR for CCSI-DL-general model after removing the pre-training vector, using one-hot as input and removing the attention mechanism. *CCSI-DL* coronavirus cross-species infection with deep learning, *spe* specific, *no pre* removing pre-training vector, *no att* removing the attention mechanism

As shown in Fig. 2A, B, the performances of three variant models of CCSI-DL-specific were not as good as those of the original model. Without the use of pre-trained DNA vector, the values of AUROC and AUPR for HCoV-229E and HCoV-HKU1 were obviously decreased (about 10–20% reduction). With the use of one-hot coding, the specific model for MERS-CoV and SARS-CoV achieved low performance. Without the use of attention mechanism, the performance for the MERS-CoV model slightly reduced while that of the HCoV-229E reduced obviously (0.91 for AUROC and 0.837 for AUPR). According to the results shown in Fig. 2A, B, it was suggested that the six virus models get the best performance with the use of pre-trained DNA vector and attention mechanism.

The performance of three variant models of general model was shown in Fig. 2C, D. The CCSI-DL-gen-nopre model had slight reduction of performance for all of the six viruses. The CCSI-DL-gen-onehot model had obviously reduction of performance for MERS-CoV, HCoV-OC43, SARS-CoV and HCoV-229E. The low performance for HCoV-229E is obvious (0.996 for AUROC and 0.967 for AUPR). Without the use of attention mechanism, all of the virus groups achieved slightly performance reduction except HCoV-229E (0.911 for AUROC and 0.875 for AUPR). The CCSI-DL-gen-noatt model had poor prediction for HCoV-229E, which only had 0.911 for AUROC and 0.875 for AUPR. According to the results shown in Fig. 2C, D, it was suggested that the CCSI-DL-general model with the use of pre-trained DNA vector and attention mechanism get the best performance.

### Advantage of artificial negative data

Compared with the genome sequences of original positive data, those of artificial negative data remain unchanged except the coding region of the spike protein. The data constrain for the spike protein is compatible with animal tests, which will increase the weight of the spike protein and will benefit for robust prediction. To show the advantage of data constrain based on biology trials, we used the original virus data to repeat the training of CCSI-DL-specific and CCSI-DL-general and predict the artificial negative data.

Table 3 shows the predictions of CCSI-DL-specific and CCSI-DL-general for the artificial negative data. It can be seen that the model trained with the initial data of coronavirus genome cannot correctly predict the artificial negative data and give positive output with thoroughly errors (about 0 for accuracy). Because the infection efficiency for the “positive” virus is vastly reduced when the encoding region of the spike protein is replaced by that of the “negative” virus, adding artificial data to the original data set for training will improve the weight of the spike protein and increase the robustness of the model.

**Table 3:** Performance of CCSI-DL-specific and CCSI-DL-general against artificial negative data

Model^a^	MERS-CoV	HCoV-OC43	HCoV-NL63	HCoV-229E	HCoV-HKU1	SARS-CoV
Specific	0.004	0	0	0.033	0	0
General	0	0	0	0	0	0

^a^Test data were wholly negative sample and the accuracy was selected as an evaluation metric. *CCSI-DL* coronavirus cross-species infection with deep learning

### Transfer learning ability of general model

The CCSI-DL-general model achieved excellent performance as shown above. To determine whether CCSI-DL-general can achieve good performance using a new data set, we fine-tuned the parameters and assessed its transfer learning ability by using a certain virus as the new data set (Dnewtrain and Dnewtest) and mixed the genome data of the other five viruses as a combined training set (Dtrain). The CCSI-DL-general model was pre-trained for 15 rounds using Dtrain, trained for 10 rounds using Dnewtrain, and evaluated using Dnewtest. The model for the evaluation of transfer leering ability was named after CCSI-DL-transfer.

**Fig. 3:**
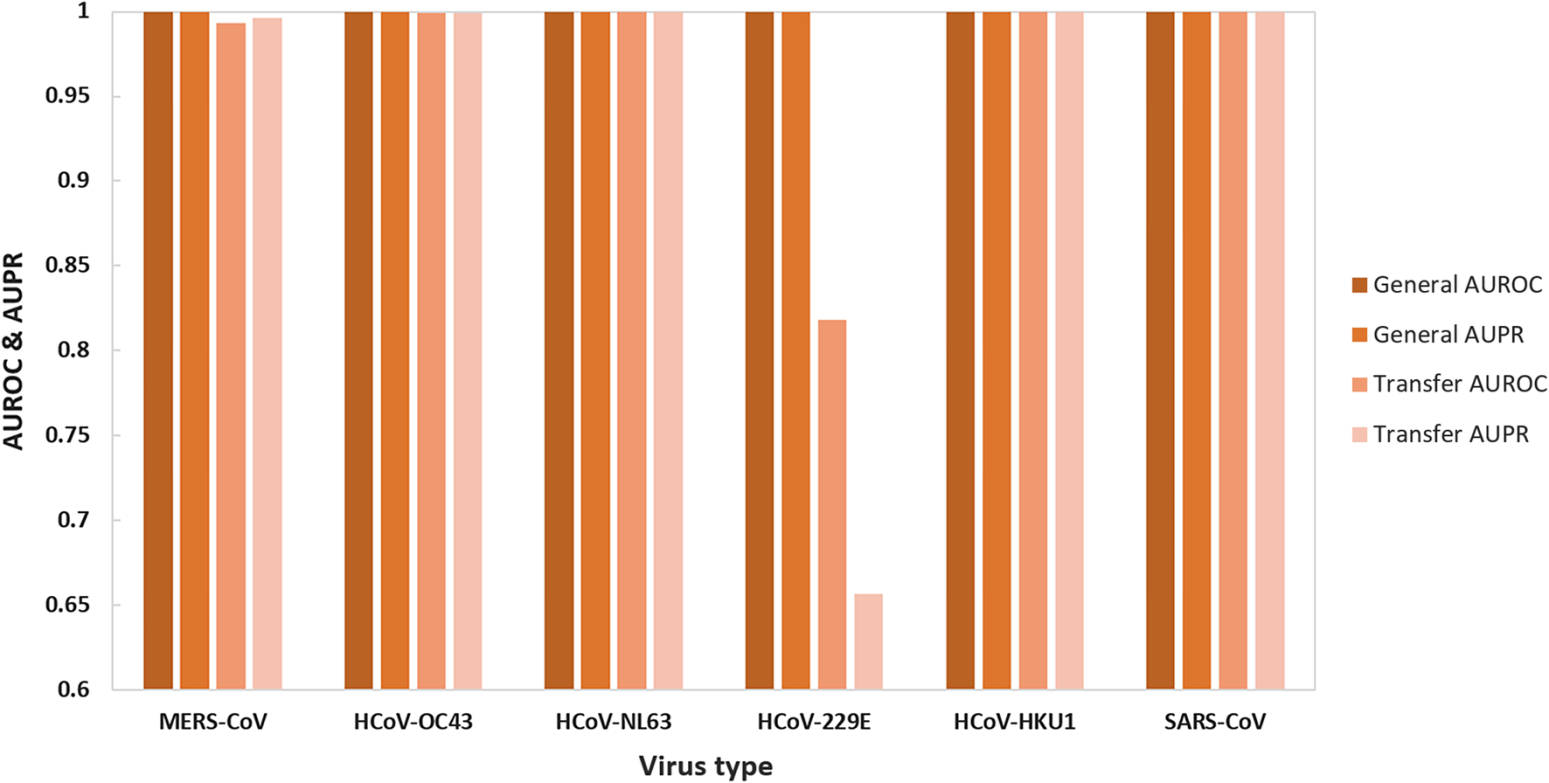
Performance of CCSI-DL-general model. Performance of the pre-trained CCSI-DL-general model transferred to a new data set. *AUROC* area under the receiver operating characteristic curve, *AUPR* area under precision–recall curve, *CCSI-DL* coronavirus cross-species infection with deep learning

As shown in Fig. 3, the performance of CCSI-DL-transfer was compared with that of CCSI-DL-general. The CCSI-DL-transfer model achieved excellent performance for HCoV-OC43, HCoV-NL63, HCoV-HKU1 and SARS-CoV (almost 1 both for AUROC and AUPR). The MERS-CoV had slight reduction of prediction performance (0.993 for AUROC and 0.996 for AUPR). However, the low performance for HCoV-229E was got with 0.818 for AUROC and 0.657 for AUPR. The main reason for the performance of HCoV-229E is about small number of virus data and high identity of genome sequence, which should be conquered with the increase of genome sequences in public database. The excellent performance for most coronavirus (average score for six groups: 0.968 for AUROC and 0.942 for AUPR) proved that the CCSI-DL-general model can be used as a pre-training model for transfer learning and will give the reasonable prediction for next pandemic pathogen.

## Discussion

Coronavirus is an animal-origin pathogen that can cause disease in humans [31, 32]. A model for phenotype identification of notorious disease is urgently needed to be developed [33, 34]. In the paper, we present a deep learning model of cross-species coronavirus infection that combines a one-dimensional convolutional neural network with a bidirectional gated recurrent unit network. This deep learning method strengths the weight of the spike protein and provides an effective model for early warning of cross-species infections.

Although the spike protein is responsible for their binding to the host cell membrane receptor and play the key role in the cross-species transmission, the synergistic effect of other viral proteins reported by biological studies of host adaption should be considered [13]. The one-dimensional convolution was used to extract the sequence features of genome sequences and the recurrent neural network with the attention mechanism was used to build the predictive model. Moreover, artificial negative data with genome recombination in the coding region of spike protein improve its weight and increase the robustness of the model.

The results about six specific models show that they are able to learn the sequence features in a single virus data set and cannot provide good predictive performances for other virus groups. However, it should be noted that the SARS-CoV model and MERS-CoV model provided well performance for each other. The similar result is suitable for the HCoV-OC43 model and the HCoV-NL63 model. The potential reason is that the similar mechanism for cross-species infection was employed.

The proposed deep learning method used the pre-trained DNA vector and attention mechanism to extract features of coronavirus genomes. Based on the evaluation of transfer learning and artificial negative data, it was proved that the general model is robust and reasonable. We used the Python programming language to create a powerful tool to benefit the surveillance for public health. Moreover, we try this tool to predict SARS-Cov-2 genome data from Brazil, United Kingdom, South Africa, and India (Positive sample; Mutant human-origin virus) and achieved 100% predictive accuracy.

The length of the genome sequence of the coronavirus is about 27–32 kb. In the paper, the long sequences of viral genomes were totally considered and divided into ten segments to increase the performance of the prediction model. However, the main limitation of the proposed method in the paper is that the interpretability of predictive results was reduced because the convolutional method was used to extract features of viral genomes and the relation between sequence features and prediction results was not obvious. The attention matrix should be used to analysis the correlation of ten genome segments. The weights of convolution network should be emphasized to master the key region in the genome sequences. Although the end-to-end model was easy to extract the feature and flexible to build the model, the development about interpretability of prediction output should be considered in the future [35], which will increase the understanding of the mechanism about cross-species coronavirus infection.

## Conclusions

We proposed a CCSI-DL model, which combines a bidirectional GRU with a one-dimensional convolution and uses the genome sequence of coronaviruses as direct input to predict the pandemic risk of human infection. We trained and tested the CSSI-DL model using single- and multi-group coronavirus genome data and achieved good performances (1 for AUROC and 1 for AUPR). Re-training experiments showed that the model has good transfer learning capabilities and the artificial negative data with genome recombination in the coding region of spike protein were correctly predicted. In contrast to traditional machine learning methods, deep learning models master the features from the whole genome of coronavirus and predict the risk of cross-species viral infections with robustness.

## Supplementary Information

**Additional file 1.** The number for the initial virus data.

**Additional file 2.** The dataset for six virus types.

**Additional file 3.** The Entry ID for the selected negative virus to recombine initial positive virus.

**Additional file 4.** The number of virus data with artificial negative data.

## Data Availability

A total of 3257 coronavirus genomes analyzed during the current study are available in the Coronavirus Genome Resource Library (https://ngdc.cncb.ac.cn/ncov/) [20]. The number for the initial virus data is provided as Additional file 1. The nomenclature for six virus types is provided as Additional file 2. The Entry ID for the selected negative virus to recombine initial positive virus is provided as Additional file 3. The number of virus data with artificial negative data is provided as Additional file 4.

## Abbreviations

CoV: Coronavirus
ICTV: International Committee on Taxonomy of Viruses
HCoV: Human coronavirus
SARS-CoV: Severe acute respiratory syndrome coronavirus
MERS-CoV: Middle East respiratory syndrome coronavirus
NLP: Natural language processing
RNN: Recurrent neural network
LSTM: Long short-term memory
GRU: Gated recurrent unit
1D: One-dimensional
ReLU: Rectified linear activation function
CCSI-DL: Coronavirus cross-species infection with deep learning
ROC: Receiver operating characteristic curve
AUROC: Area under the receiver operating characteristic curve
AUPR: Area under precision–recall curve
